# The role of hemoadsorption in cardiac surgery – a systematic review

**DOI:** 10.1186/s12872-024-03938-4

**Published:** 2024-05-18

**Authors:** Marijana Matejic-Spasic, Sandra Lindstedt, Guillaume Lebreton, Omer Dzemali, Piotr Suwalski, Thierry Folliguet, Stephan Geidel, Robert J. M. Klautz, Christophe Baufreton, Ugolino Livi, Serdar Gunaydin, Efthymios N. Deliargyris, Daniel Wendt, Matthias Thielmann

**Affiliations:** 1 CytoSorbents Europe GmbH, Berlin, Germany; 2 Department of Cardiothoracic Surgery and Transplantation, Skane University Hospital, Lund, Sweden; 3 Thoracic and Cardiovascular Surgery Department, Pitié-Salpêtrière University Hospital, Paris, France; 4 Department of Cardiac Surgery, University Hospital Zurich, Zurich, Switzerland; 5 Department of Cardiac Surgery, City Hospital Triemli, Zurich, Switzerland; 6 Department of Cardiac Surgery, Central Clinical Hospital of the Ministry of the Interior and Administration, Warsaw, Poland; 7 Department of Cardiac Surgery, Henri Mondor Hospital, Paris, France; 8 Department of Cardiac Surgery, Asklepios Klinik St. Georg, Hamburg, Germany; 9 Department of Cardio-Thoracic Surgery, University Medical Center, Leiden, The Netherlands; 10 Department of Cardiovascular and Thoracic Surgery, University Hospital, Angers, France; 11 Department of Cardiothoracic Surgery, University Hospital, Udine, Italy; 12 Department of Cardiovascular Surgery, University of Health Sciences, Ankara City Hospital Campus, Ankara, Turkey; 13 CytoSorbents Inc, Princeton, NJ USA; 14 Department of Thoracic- and Cardiovascular Surgery, Westgerman Heart and Vascular Center, University Duisburg-Essen, Essen, Germany

**Keywords:** Cardiac surgery, Hemoadsorption, Hyperinflammation, Blood purification, CytoSorb, Infective endocarditis, Aortic surgery, Heart transplantation, ECMO

## Abstract

**Background:**

Extracorporeal blood purification has been widely used in intensive care medicine, nephrology, toxicology, and other fields. During the last decade, with the emergence of new adsorptive blood purification devices, hemoadsorption has been increasingly applied during CPB in cardiac surgery, for patients at different inflammatory risks, or for postoperative complications. Clinical evidence so far has not provided definite answers concerning this adjunctive treatment. The current systematic review aimed to critically assess the role of perioperative hemoadsorption in cardiac surgery, by summarizing the current knowledge in this clinical setting.

**Methods:**

A literature search of PubMed, Cochrane library, and the database provided by CytoSorbents was conducted on June 1st, 2023. The search terms were chosen by applying neutral search keywords to perform a non-biased systematic search, including language variations of terms “cardiac surgery” and “hemoadsorption”. The screening and selection process followed scientific principles (PRISMA statement). Abstracts were considered for inclusion if they were written in English and published within the last ten years. Publications were eligible for assessment if reporting on original data from any type of study (excluding case reports) in which a hemoadsorption device was investigated during or after cardiac surgery. Results were summarized according to sub-fields and presented in a tabular view.

**Results:**

The search resulted in 29 publications with a total of 1,057 patients who were treated with hemoadsorption and 988 control patients. Articles were grouped and descriptively analyzed due to the remarkable variability in study designs, however, all reported exclusively on CytoSorb^®^ therapy. A total of 62% (18/29) of the included articles reported on safety and no unanticipated adverse events have been observed. The most frequently reported clinical outcome associated with hemoadsorption was reduced vasopressor demand resulting in better hemodynamic stability.

**Conclusions:**

The role of hemoadsorption in cardiac surgery seems to be justified in selected high-risk cases in infective endocarditis, aortic surgery, heart transplantation, and emergency surgery in patients under antithrombotic therapy, as well as in those who develop a dysregulated inflammatory response, vasoplegia, or septic shock postoperatively. Future large randomized controlled trials are needed to better define proper patient selection, dosing, and timing of the therapy.

**Supplementary Information:**

The online version contains supplementary material available at 10.1186/s12872-024-03938-4.

## Background

Major surgery-related trauma and cardiopulmonary bypass (CPB) itself (induced by artificial surface contact), are associated with hyperinflammation, formerly described in the literature as SIRS – Systemic Inflammatory Response Syndrome. Despite recent advancements in surgical and anesthetic techniques, open cardiac surgery *per se* still carries a significant risk for morbidity and mortality [1]. Even with recent developments towards minimally invasive techniques, cardiac surgery using CPB is still the current “gold standard”. Moreover, the complexity of cardiac surgery will further increase due to the aging population, frailty, and many comorbidities. Despite recent advantages in myocardial protection for several hours, systemic pathological inflammation derived from extracorporeal circulation might still occur [2].

Various measures have been introduced to prevent or treat dysregulated inflammatory response in cardiac surgery and reduce its serious harm. However, a single approach cannot block multiple (severe) inflammation pathways. Extracorporeal blood purification techniques have been widely used in intensive care medicine, nephrology, toxicology, and other fields. During the last decade, with the emergence of new adsorptive blood purification devices, hemoadsorption has been increasingly applied during CPB in cardiac surgery, for patients at different inflammatory risks, or for postoperative complications [2].

This review aims to critically assess the role of hemoadsorption in cardiac surgery, by summarizing the results of published studies conducted in this clinical setting in the last decade.

## Methods

The scope of the current literature search was to identify all relevant studies to summarize the current level of evidence concerning the use of hemoadsorption in the field of cardiac surgery. The search terms were chosen by applying neutral search keywords to perform a non-biased systematic search and retrieve all available data.

### Literature search strategy

A comprehensive literature search of the online database of the United States National Library of Medicine (PubMed), the Cochrane Library, and the database provided by CytoSorbents (01.1.2010–01.6.2023). In the literature screening and selection process, we followed the principles derived from the Preferred Reporting Items for Systematic Reviews and Meta-Analyses (PRISMA) statement [3], to preserve an objective approach (Additional file 1: PRISMA 2020 Checklist). A search of databases was made on June 1^st^, 2023, using the following key search words: “cardiac surgery” OR “cardiothoracic surgery” AND “hemoadsorption” OR “hemoadsorbtion” OR “hemadsorption” OR “hemadsorbtion” OR “haemoadsorption” OR “haemoadsorbtion” OR “haemadsorption” OR “haemadsorbtion”. Abstracts were considered for inclusion if they were written in English and published within the last ten years.

### Eligibility criteria

Publications were eligible for assessment if reporting on original data from any type of study, excluding case reports, in which hemoadsorptive device was investigated during or after cardiac surgery.

### Data extraction and critical appraisal

The most important details about all the studies are presented in a tabular view (Additional file 2: Evidence table). Outcomes related to the intervention were deemed eligible to be included if statistically significant differences were found. For defined primary outcomes, statistically non-significant findings were also included. Considering secondary outcomes, if the difference was not statistically significant but still striking, or the observed trend was evident, such results were added and described as “notable”. Three researchers (P.S., M.T., C.B.) independently assessed these results and the final agreement on the inclusion of notable differences in the analysis was reached by consensus. Primary outcomes were highlighted if defined as such in the respective study. In studies without statistical analysis, findings highlighted in respective articles were copied.

## Results

**Fig. 1:**
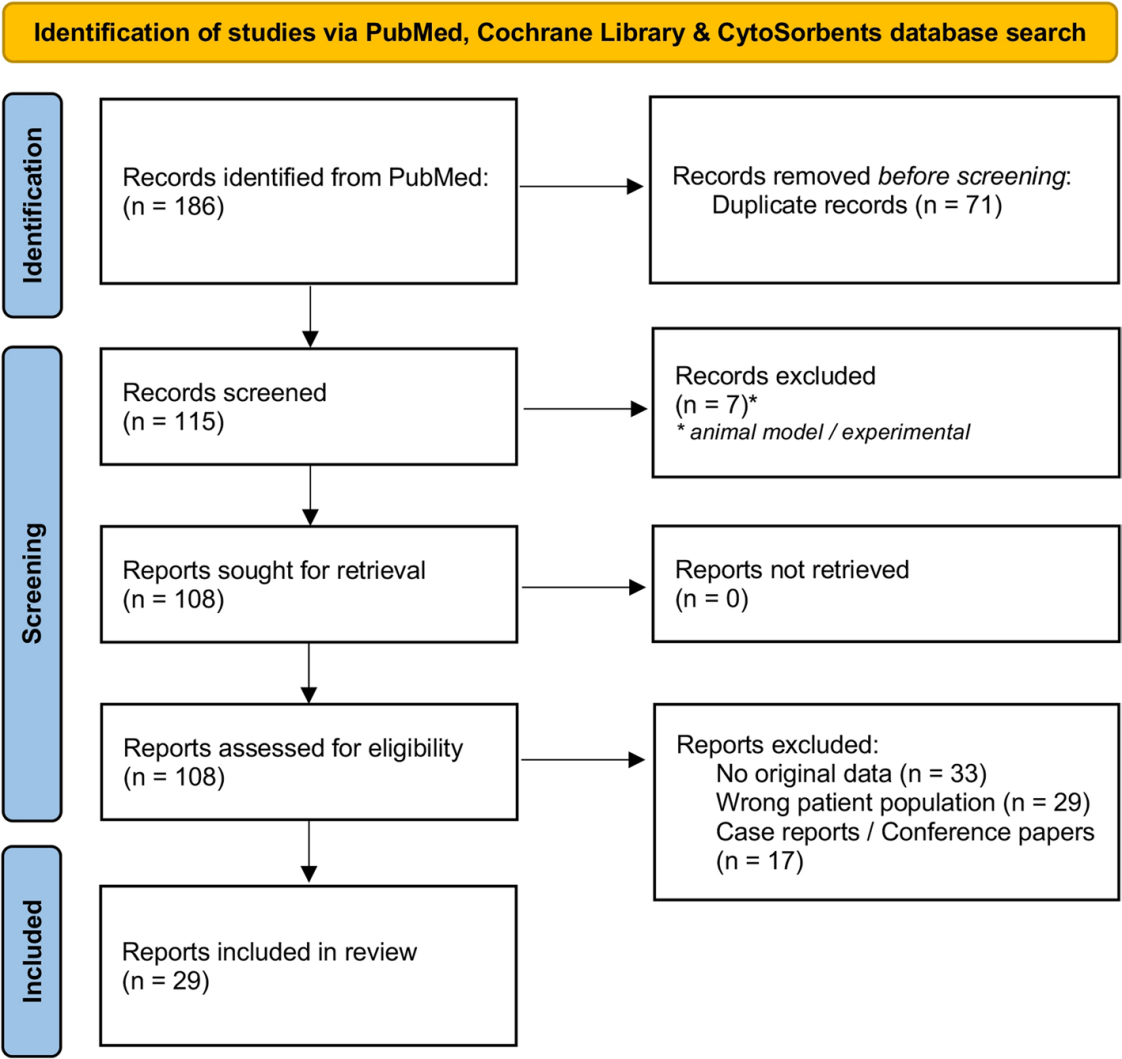
Systematic literature search flowchart

The search resulted in 186 hits in total. After duplicates were removed (*n* = 71), the remaining abstracts were screened and an additional seven were excluded (details given within the flowchart, Fig. 1). Furthermore, 108 reports were assessed out of which 79 were deemed ineligible—29 included the wrong patient population (26 studies not associated with cardiac surgery and 3 publications reporting on hemoadsorption utilized for the removal of antithrombotic drugs – a topic already comprehensively covered elsewhere [4]), 33 articles contained no original data, and 17 were case reports or conference papers.

### Quantity of evidence

The literature search resulted in a total of 29 publications that investigated hemoadsorption in cardiac surgery. The technology described in all of them was exclusively the CytoSorb^®^ device (CytoSorbents Inc., Princeton, NJ, USA). The summary of retrieved articles in chronological order, and intervention-reported outcomes, are given in the Evidence table (Additional file 2). This review included a total of 1,057 patients who were treated with hemoadsorption and 988 control patients (subjects from post hoc analyses excluded). Considering the fact that several studies were conducted in the same centers and during overlapping periods, a portion of the above patients may be duplicates, however, it was impossible to identify them and exclude them from the present analysis. Appropriate notes are, therefore, included in respective sections.

### Assessment of endpoints

The variability in study design, patient population, and reported endpoints was vast. Therefore, we decided to report the results of this search in systematic subgroups according to the underlying clinical indication or main surgical procedure. A separate subgroup for patients treated with hemoadsorption in the postoperative cardio-surgical period was also created. The most significant proportion of publications reported on intraoperative hemoadsorption utilization (25/29, 86.2%), including surgery for infective endocarditis (10/29, 34.5%), complex elective cardiothoracic surgery with prolonged CPB time (10/29, 34.5%), aortic surgery (3/29, 10.3%), left ventricular assist device implantation (1/29, 3.4%), and heart transplantation (1/29, 3.4%). The remaining four publications (4/29, 13.8%) reported on the postoperative use of hemoadsorption therapy adjacent to other extracorporeal blood purification techniques such as continuous renal replacement therapy (CRRT) or extracorporeal membrane oxygenation (ECMO). The most critical details from the grouped publications are given in the respective sections below. Finally, we assessed the safety of this technique by analyzing device-related adverse events outlined within the obtained literature.

### Infective endocarditis

Between 2017 and 2023, 10 published studies investigated the use of hemoadsorption in infective endocarditis surgery (Table 1). Three were randomized controlled trials (RCTs), one case series, and six were comparative retrospective case-controlled studies with three employing dedicated statistical methods for case/control matching. The total cohort of this subgroup comprised 497 patients treated with hemoadsorption and 608 controls (a portion of subjects are likely duplicates, however, the identification and exclusion could not be performed).

**Table 1:** Evidence overview – infective endocarditis associated valve surgery

No	Study	Study design	Device	Interventions	Controls	Results of the intervention
1	2017 Träger, K., et al. [5]	Retrospective Case-control^a^	CytoSorb^®^ (intra-op on CPB)	39	28	Notably: - shorter ICU length of stay Pre- *vs* post-treatment: - reduction in vasopressor demand - reduction of cytokine IL-6 & IL-8 levels Rapid normalization of: - lactate levels and base excess - MAP
2	2019 Kühne, L. U., et al. [6]	Retrospective Case-control^a^	CytoSorb^®^ (intra-op on CPB + post-op on CRRT)	10	10	Despite a more pronounced disease severity in patients who received the therapy both intra- and postoperatively, compared to those with only intra-op hemoadsorption, equal post-op: - vasopressors - CRP - lactate - ventilator time
3	2020 Haidari, Z., et al. [7]	Retrospective Case–control	CytoSorb^®^ (intra-op on CPB)	30	28	Significantly: - lower incidence of sepsis (primary outcome) - lower sepsis-related mortality (primary outcome) - reduced vasopressor requirements - higher SVR Notably lower overall 30-day mortality
4	2021 Asch, S., et al. [8]	Prospective RCT	CytoSorb^®^ (intra-op on CPB + post-op on CRRT)	10	10	Significantly: - higher vasopressor need - higher volume of fluids - longer ICU length of stay No significant difference in cytokine levels – primary outcome (IL-6, TNF-α) CRP and PCT baseline levels were significantly higher in the intervention group, equalizing after surgery
5	2021 Santer, D., et al. [9]	Retrospective Case–control (IPTW)	CytoSorb^®^ (intra-op on CPB)	41	200	Significantly: - higher norepinephrine and milrinone demand - more RBC and PLT transfusions - higher incidence of reoperations for bleeding - prolonged hospitalization No significant difference in in-hospital mortality – primary outcome
6	2022 Diab, M., et al. [10]	Prospective RCT	CytoSorb^®^ (intra-op on CPB)	138	144	Significantly: - lower levels of cytokines IL-1β, IL-18 (in the first 25 *vs* 25 patients) No significant difference in SOFA score change (primary outcome), as well as in clinical outcomes
7	2022 Haidari, Z., et al. [11]	Retrospective Case–control (PSM)	CytoSorb^®^ (intra-op on CPB)	35	35	Significantly: - lower sepsis-related mortality (primary outcome) - reduced vasopressor demand - higher SVRI - faster SOFA score normalization - lower respiratory failure rate No significant difference in the postoperative sepsis incidence and in-hospital mortality – primary outcomes
8	2022 Holmen, A., et al. [12]	Prospective RCT	CytoSorb^®^ (intra-op on CPB)	10	9	Significantly: - fewer transfusions (RBC, PLT, FFP) Notably: - lower and shorter norepinephrine demand (primary outcome) - lower creatinine levels - lower chest-tube drainage volume - shorter ventilator time
9	2022 Kalisnik, J. M., et al. [13]	Retrospective Case–control (PSM)	CytoSorb^®^ (intra-op on CPB)	99	99	Significantly: - lower incidence of sepsis (primary outcome) - lower sepsis-related mortality (primary outcome) - lower CRP levels - fewer transfusions (RBC & FFP) - lower WBC counts - higher hemoglobin level Notably lower in-hospital mortality
10	2023 Haidari, Z., et al. [14]	Retrospective Case–control	CytoSorb^®^ (intra-op on CPB)	75	55	Significantly: - decreased VIS (primary outcome) - lower incidence of sepsis-related mortality - lower 30-day & 90-day mortality - lower incidence of renal failure requiring hemodialysis Notably: - lower incidence of revisions for bleeding

*CPB* Cardiopulmonary bypass, *IL* Interleukin, *MAP* Mean arterial pressure, *ICU* Intensive care unit, *CRRT* Continuous renal replacement therapy, *CRP* C reactive protein, *SVR* Systemic vascular resistance, *RCT* Randomized controlled trial, *TNF* Tumor necrosis factor, *PCT* Procalcitonin, *IPTW* Inverse probability treatment weighting, *RBC* Red blood cells, *PLT* Platelets, *SOFA* Sequential organ failure assessment, *PSM* Propensity score matching, *SVRI* Systemic vascular resistance index, *FFP* Fresh frozen plasma, *WBC* White blood cells, *VIS* Vasoactive-inotropic score

^a^Statistical analysis for significant differences was not performed

In all studies, CytoSorb® was used intraoperatively by integration into the CPB-circuit, but in the Kühne et al. case series [6] and the small RCT from Asch et al. [8], hemoadsorption therapy was additionally continued postoperatively during the intensive care unit (ICU) period.

The most frequently reported outcome was vasopressor requirements, and in all but two studies [10, 13], a significant reduction (where statistical analysis was performed) in vasopressor drug demand was observed with intraoperative hemoadsorption. Cytokine reductions with CytoSorb^®^ were confirmed in two studies [5, 10], while significantly lower postoperative sepsis-related mortality with hemoadsorption was observed in four studies [7, 11, 13, 14]. Out of these four, one recent study showed a significantly reduced 30- and 90-day mortality in selected patients suffering from *Staphylococcus aureus*-derived infective endocarditis [14].

Other reported outcomes varied according to each study and are discussed in more detail in the Discussion part of this review.

### Elective complex cardiac surgery

The search yielded 10 articles reporting on studies that enrolled patients undergoing elective, but complex cardiac surgical procedures (Table 2). Eight of the 10 studies were RCTs, six original and two *post hoc* subgroup analyses from the oldest RCT in this group from Bernardi et al. [15], while the remaining two studies were a prospective case series [16], and a retrospective comparative study [17]. The total cohort of this subgroup comprised 109 patients treated with hemoadsorption and 131 controls (subjects from *post hoc* analyses excluded) in the period 2016–2022.

**Table 2:** Evidence overview – elective complex cardiac surgery (various procedures with prolonged CPB)

No	Study	Study design	Device	Interventions	Controls	Results of the intervention
1	2016 Bernardi, M. H., et al. [15]	Prospective RCT	CytoSorb^®^ (intra-op on CPB)	19	18	No significant difference in primary outcome—cytokine levels (IL-1ß, IL-6, IL-18, TNF-⍺), except for IL-10
2	2019 Bernardi, M. H., et al. [18]	Prospective RCT (*post hoc* subgroup analysis of No. 1)	CytoSorb^®^ (intra-op on CPB)	17	18	Significantly: - higher haptoglobin (primary outcome) - lower LDH No significant difference between the groups in levels of plasma-free hemoglobin (primary outcome) and total bilirubin
3	2019 Garau, I., et al. [19]	Prospective RCT	CytoSorb^®^ (intra-op on CPB)	20	20	Significantly: - lower cytokine levels (IL-8, TNF-⍺)—primary outcome - higher CI No significant difference in levels of IL-6 (primary outcome)
4	2019 Gleason, T. G., et al. [20]	Prospective RCT	2 CytoSorb^®^ (intra-op on CPB)	23	23	Significant reduction in: - plasma-free hemoglobin (primary outcome) - activated complement C3a & C5a
5	2019 Poli, E. C., et al. [21]	Prospective RCT	CytoSorb^®^ (intra-op on CPB)	15	15	No significant difference in primary outcome—cytokine levels (IL-1a, IL-1b, IL-2, IL-4, IL-5, IL-6, IL-10, TNF-α, IFN-γ, MCP-1), as well as in clinical outcomes Significantly lower activity of coagulation factors II and XII
6	2019 Wagner, R., et al. [22]	Prospective RCT	CytoSorb^®^ (intra-op on CPB)	15	13	Significantly higher level of miRNA-133 – primary outcome No significant differences in levels of miRNA-1, miRNA-126, and miRNA-223 (primary outcomes), as well as in clinical outcomes
7	2019 Taleska-Stupica, G., et al. [23]	Prospective RCT	CytoSorb^®^ (intra-op on CPB)	20	20 & 20^a^	Significantly: - higher CD64 and CD163 antigen expression on immune cells - increased activated complement C5a (primary outcomes) No significant difference in primary outcome—cytokine levels (TNF-*α*, IL-1*β*, IL-6, IL-8, and IL-10)
8	2020 Wisgrill, L., et al. [24]	Prospective RCT (*post hoc* subgroup analysis of No. 1)	CytoSorb^®^ (intra-op on CPB)	9	9	No significant differences in circulating microvesicles, apoptotic body counts and kinetics
9	2021 Hohn, A., et al. [16]	Prospective Case series (part of the ongoing RECCAS^b^ study)	CytoSorb^®^ (intra-op on CPB)	15	/	Significant, pre- *vs* post-adsorber: - reduction of heparan sulphate - increase of hyaluronan
10	2022 Manohar, M., et al. [17]	Retrospective Case–control	CytoSorb^®^ (intra-op on CPB)	23	29	Significantly lower: - increase of VIS from pre- to postoperative value (primary outcome) Notably lower: - in-hospital mortality

*CPB* Cardiopulmonary bypass, *RCT* Randomized controlled trial, *IL* Interleukin, *TNF* Tumor necrosis factor, *LDH* Lactate dehydrogenase, *CI* Cardiac index, *IFN* Interferon, *MCP* Monocyte chemoattractant protein, *miRNA* Micro ribonucleic acid, *VIS* Vasoactive-inotropic score

^a^Three groups, 20 given intraoperative methylprednisolone, 20 intraoperative CytoSorb*®*, 20 controls; results shown for comparison between hemoadsorption and controls

^b^German Clinical Trials Register number DRKS00007928 (Date of registration August 3rd, 2015)

Procedures included open valve surgery, coronary artery bypass grafting (CABG)—isolated and combined [15, 18, 19, 24], or various other cardiac surgery operations with prolonged CPB times (> 90 min) [16, 17, 20, 21, 23], including one study that enrolled patients who underwent combined aortic root and valve surgery [22]. Hemoadsorption was used exclusively intraoperatively in all studies, however in one study two adsorbers in parallel connection within the CPB circuit were used [20].

There was no significant reduction of circulating cytokine levels in two RCTs [15, 21], while one RCT [19] detected significant reductions in cytokine levels in the hemoadsorption group. Two studies [18, 20] had conflicting results about levels of plasma-free hemoglobin (pfHb) – Bernardi et al. [18] found no reduction of pfHb within the intervention arm, while Gleason et al. [20] did. The former additionally found significant differences in markers of hemolysis such as haptoglobin and lactate dehydrogenase.

The Discussion section further elaborates on reported secondary outcomes and their clinical relevance.

### Aortic surgery

Three studies since 2019 investigated the effect of adjunctive intraoperative use of CytoSorb^®^ in aortic surgery (Table 3). The most extensive study [25] included various surgical interventions involving the thoracic aorta while patients were in hypothermic circulatory arrest, comprising elective and acute procedures. Of note, the investigators analyzed complex aortic surgery patients including selective cerebral perfusion with hypothermic circulatory arrest. A small pilot study from India [26] mainly included elective aortic root replacements and a pilot RCT from Germany [27] enrolled patients who underwent open thoracoabdominal aortic aneurysm (TAAA) repair on CPB. This subgroup contains 186 patients treated with hemoadsorption who were compared to 193 control patients.

**Table 3:** Evidence overview – aortic surgery

No	Study	Study design	Device	Interventions	Controls	Results of the intervention
1	2019 Saller, T., et al. [25]	Retrospective Case–control (PSM) Procedure: Aortic surgery with hypothermic circulatory arrest	CytoSorb^®^ (intra-op on CPB)	168	168	Significantly: - lower requirement for intraoperative norepinephrine - less pRBC & FFP transfusions - higher requirement for PCC Notably: - improved acid–base balance - lower intraoperative mortality
2	2021 Mehta, Y., et al. [26]	Retrospective Case–control Procedure: Ascending aorta replacement	CytoSorb^®^ (intra-op on CPB)	8	8	Significantly: - lower IL-6 (primary outcome) - requirement for norepinephrine - ICU and hospital stay Notably: - improved PaO_2_/FiO_2_ ratio - duration of mechanical ventilation No significant differences in primary outcomes PCT, WBC count, and CRP
3	2023 Doukas, P., et al	Prospective RCT Procedure: Thoracoabdominal aortic repair	CytoSorb^®^ (intra-op on CPB)	10	17	Significantly: - lower incidence of severe ARDS Notably: - shorter duration of mechanical ventilation

*PSM* Propensity score matching, *CPB* Cardiopulmonary bypass, *pRBC* Packed red blood cells, *FFP* Fresh frozen plasma, *PCC* Prothrombin complex concentrate, *IL* Interleukin, *ICU* Intensive care unit, *PCT* Procalcitonin, *WBC* White blood cells, *CRP* C reactive protein, *RCT* Randomized controlled trial, *ARDS* Acute respiratory distress syndrome

Overall, less need for vasopressor therapy and blood product transfusions were observed compared to controls, and there was a lower incidence of acute respiratory distress syndrome (ARDS) in the TAAA patients. Mechanical ventilation-related outcomes were notably better within the intervention groups.

### Heart transplantation

Nemeth et al. [28] conducted an observational pilot study in the setting of orthotopic heart transplantation (HTx). The results were published in 2018. The primary outcome was defined as hemodynamic stability and vasopressor demand during the first 48 h postoperatively and the magnitude of postoperative inflammatory response described by the kinetics of procalcitonin (PCT) and C reactive protein (CRP). Patients undergoing orthotopic HTx who received CytoSorb^®^ intraoperatively were compared to propensity score-matched controls (16 *vs.* 16). In the postoperative period, a significant difference in the need for vasopressor was found between the groups. The control patients required more norepinephrine and terlipressin. The dynamics of PCT and CRP did not vary between the groups. In addition, the incidence of primary graft failure was significantly lower in the hemoadsorption group, and these patients required mechanical circulatory support and renal replacement therapy less frequently. Notably, lower lactate levels and reoperations for bleeding were observed in the hemoadsorption group, which was associated with a shorter duration of mechanical ventilation time and ICU stay. These benefits translated to lower 30-day mortality in intraoperative hemoadsorption patients significantly. No device-related adverse events were reported (Evidence table, Additional file 2).

### LVAD

One article, published in 2022, reported on hemoadsorption use intraoperatively during left ventricular assist device (LVAD) implantation [29]. A propensity score-matched comparison of 72 patients who received intraoperative hemoadsorption and 40 who did not, revealed a significantly higher incidence of respiratory failure within the intervention group, and higher rates of prolonged mechanical ventilation and tracheostomy. The primary outcome—overall survival after LVAD implantation, was comparable between the groups. Adverse events were reported equally in both groups (Evidence table, Additional file 2).

### Postoperative management

Four of the 29 articles in this review, published between 2016 and 2021, reported using hemoadsorption in the postoperative period (Table 4). All were retrospective single cohort studies evaluating the use of CytoSorb^®^ in patients with evidence of ongoing hyperinflammation, or patients who required mechanical circulatory support for septic shock and multiorgan failure. The device was most frequently integrated via continuous renal replacement therapy (CRRT), followed by integration in veno-arterial extracorporeal membrane oxygenation (vaECMO) circuits or other platforms. In aggregate, 177 patients were included.

**Table 4:** Evidence overview – post-cardiac surgery complications

No	Study	Study design	Device	Interventions	Controls	Results of the intervention
1	2016 Träger, K., et al. [30]	Retrospective Case series Population: Post-op hyperinflammation (SIRS)	CytoSorb^®^ (post-op on CRRT)	16	/	Pre- *vs* post-treatment: - IL-6 & IL-8 reductions - less vasopressor demand - improved MAP & CI - improved SOFA score - reduction of lactate levels - normalized base excess - shorter ICU length of stay
2	2019 Calabro, M. G., et al. [31]	Retrospective Case series Population: Post-op MOF (cardiac-related)	CytoSorb^®^ (post-op on various platforms)	40	/	Pre- *vs* post-treatment, significant: - reduction of bilirubin - reduction of lactate levels - reduction of CPK & LDH - lower VIS SOFA-predicted *vs* observed ICU mortality: 80% *vs* 55%
3	2020 Träger, K., et al. [32]	Retrospective Case series Population: Post-op MOF (cardiac-related)	CytoSorb^®^ (on vaECMO)	23	/	Pre- *vs* post-treatment, significant: - IL-6 reduction - norepinephrine reduction - reduction of lactate levels - normalized base excess
4	2021 Boss, K., et al. [33]	Retrospective Case series Population: Post-op septic shock	CytoSorb^®^ (post-op on CRRT)	98	/	Pre- *vs* post-treatment, significantly: - decreased VIS - reduced lactate levels - reduced SOFA & APACHE II scores SOFA/APACHE II-predicted *vs* observed mortality: 77/73% *vs* 59%

*SIRS* Systemic inflammatory response syndrome, *CRRT* Continuous renal replacement therapy, *IL* Interleukin factor, *MAP* Mean arterial pressure, *CI* Cardiac index, *SOFA* Sequential organ failure assessment, *ICU* Intensive care unit, *MOF* Multiorgan failure, *CPK* Creatine phosphokinase, *LDH* Lactate dehydrogenase, *VIS* Vasoactive inotropic score, *vaECMO* Veno-arterial extracorporeal membrane oxygenation, *APACHE* Acute physiology and chronic health evaluation

Results of the intervention are presented as pre- *versus* post-treatment. Hemoadsorption correlated with decreased vasoactive-inotropic score (VIS) [31, 33] and reductions in cytokines [30, 32].

Additionally, two studies compared the actual death rate with the expected mortality based on the standardized Sequential Organ Failure Assessment (SOFA) and/or Acute Physiology and Chronic Health Evaluation II (APACHE II) prediction scores. In both cohorts, observed mortality was notably lower than SOFA-predicted – 55% *vs*. 80% [31] and SOFA/APACHE II-predicted 59% *vs.* 77/73% [33], respectively.

### Safety

The safety profile of CytoSorb^®^ can be assessed by analyzing reported device-related adverse events (Evidence table, Additional file 2). In 18 / 29 retrieved articles authors reported that unanticipated adverse events associated with hemoadsorption were not observed (62%). Six studies (21%) reported equal rates of clinical adverse events between the intervention and control groups but did not mention device-relatedness, and five publications (17%) did not report adverse events. In aggregate, no serious adverse device-related events were reported in the included articles.

## Discussion

This systematic review on hemoadsorption use in cardiac surgery has summarized the available published evidence on using CytoSorb^®^. The heterogeneity of the studies prevents the performance of a systematic meta-analysis of reported outcomes, hence the results are presented descriptively. Although other hemoadsorptive technologies may be used in cardiac surgery, our holistic literature search only identified published evidence for CytoSorb^®^.

### Hyperinflammation after Cardiopulmonary Bypass

The terms SIRS [34] and “cytokine storm” [35] were introduced in the early 1990s and are also frequently used to describe the underlying pathophysiological process to explain the problematic postoperative course underscored by vasoplegia that occurs in some patients after cardiac surgery [36]. Several triggers and pathophysiological mechanisms have been proposed [37] and there are ongoing efforts to identify novel solutions to address this serious complication. Treatment options mostly mirror those utilized in septic shock due to the comparable central role of the dysregulated immune response. Blood purification to remove elevated levels of cytokines and other inflammatory mediators has emerged as an attractive option to stop the vicious circle of auto-amplifying systemic hyperinflammation often leading to vasoplegic shock [38] and multiorgan failure. The CytoSorb^®^ adsorber is the most researched device for attenuating hyperinflammation and for rebalancing the dysfunctional immune response [39, 40].

### CytoSorb^®^ therapy

**Fig. 2:**
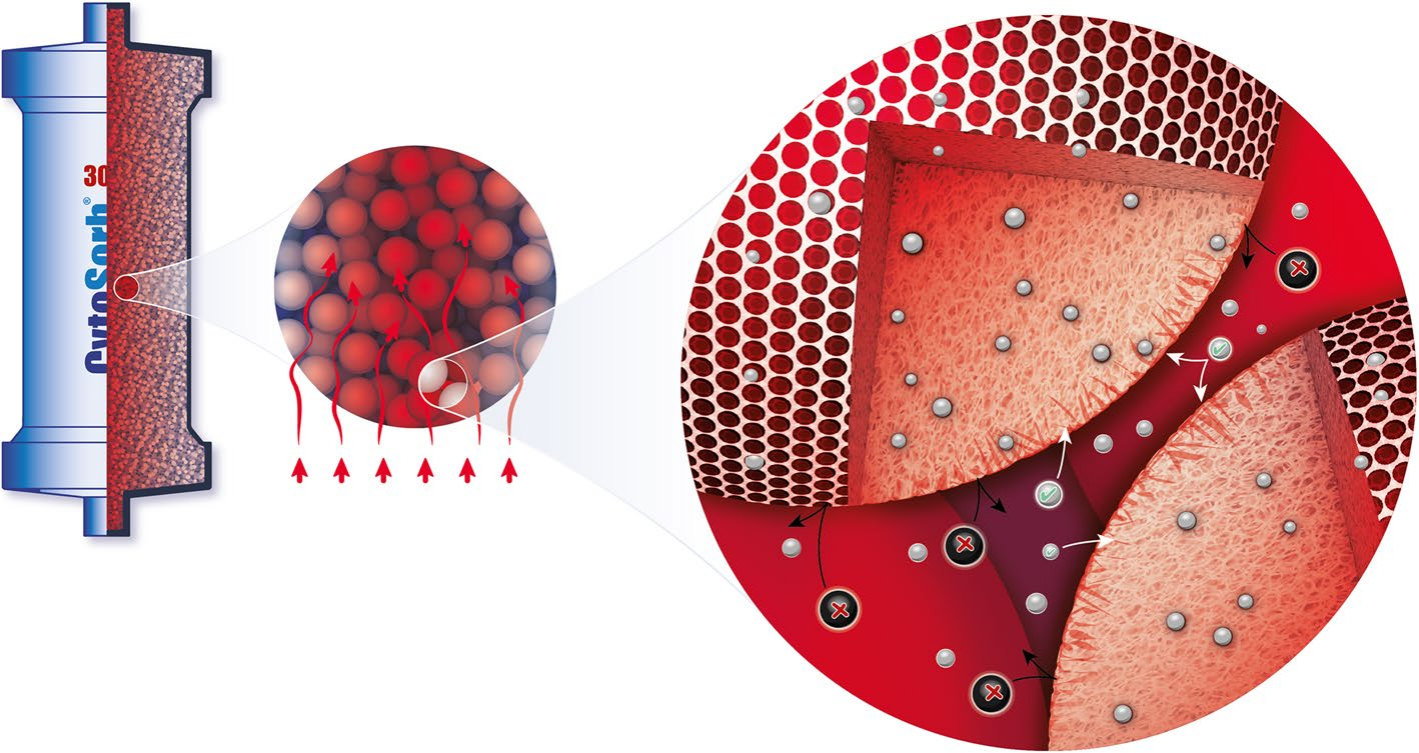
CytoSorb^®^ polymer bead technology

CytoSorb^®^ therapy is a blood purification technique based on the hemoadsorption of hydrophobic molecules of up to approximately 60 kDa of molecular weight (Fig. 2). It is easily integrated into extracorporeal circuits (CPB, (C)RRT, ECMO, etc.) and is CE mark approved for the removal of cytokines, bilirubin, myoglobin, ticagrelor, and rivaroxaban [41].

**Fig. 3:**
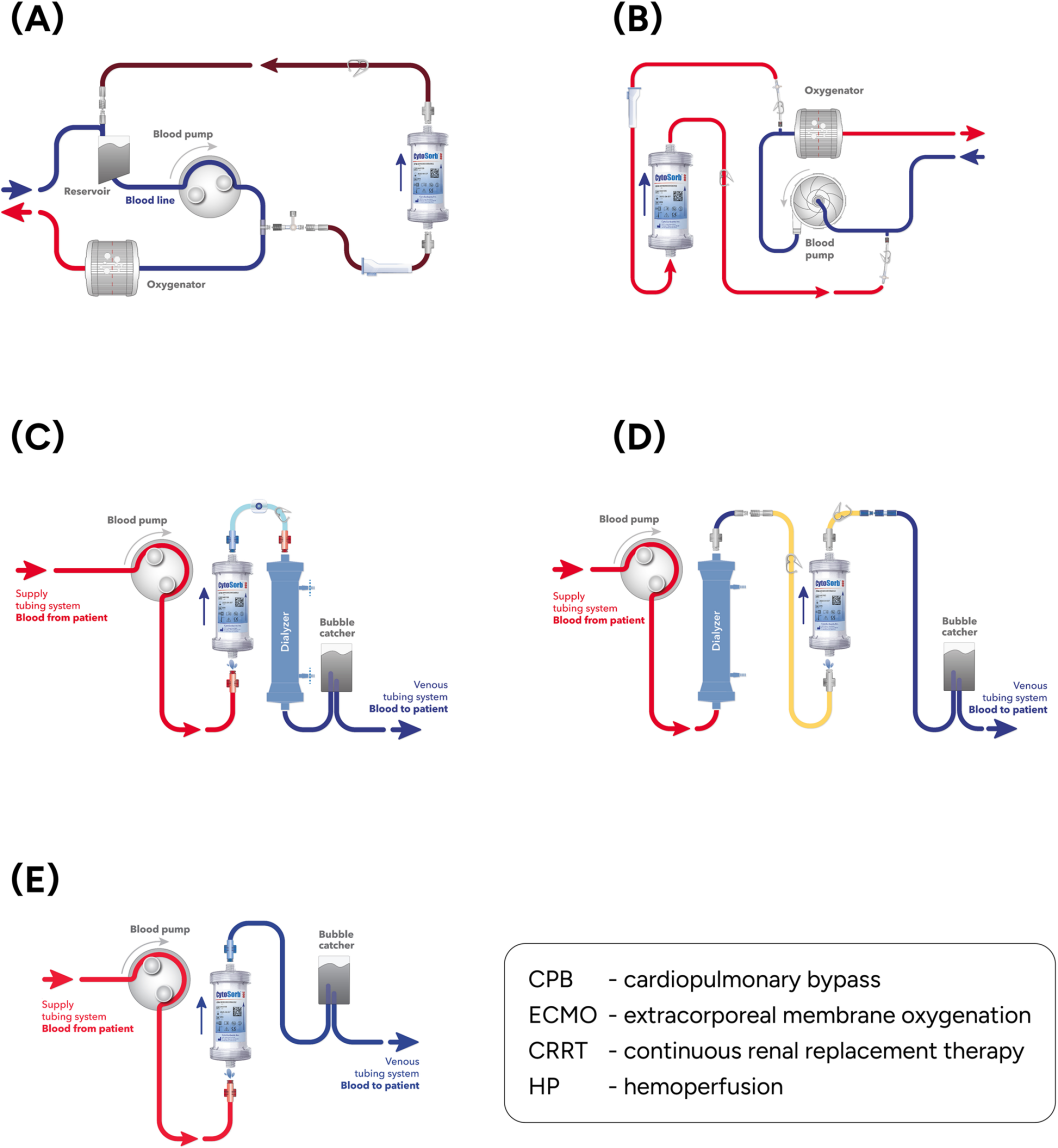
Installation scheme of CytoSorb^®^ device within (**A**) CPB, **B** ECMO, **C** CRRT – pre-filter position, **D** CRRT – post-filter position, and **E** stand-alone HP mode

In cardiac surgery, it is predominantly used intraoperatively, installed in a by-pass circuit, providing hemoadsorption of undesirable molecules within the duration of CPB (Fig. 3A), with an aim to prevent perioperative complications induced by inflammatory mediators or antithrombotics. Postoperatively, it may be similarly utilized within ECMO circuit (Fig. 3B), adjunctive to hemodialysis (Fig. 3C & D), or in a simple hemoperfusion (HP) mode as a stand-alone blood purification technique (Fig. 3E).

**Fig. 4:**
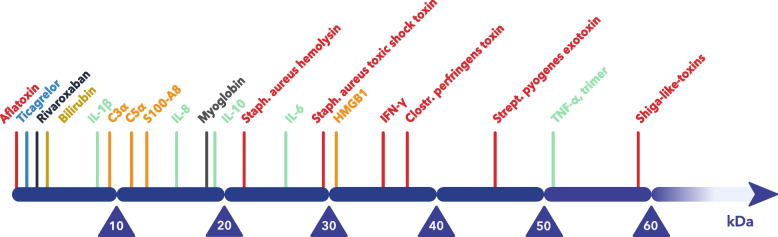
CytoSorb^®^ adsorption range

According to the Instruction for Use [41], setup and management of intraoperative hemoadsorption is uncomplicated as the adsorber can be prepared in under 10 min. The absence of safety concerns is supported by the findings of published RCTs like the REMOVE [10] or REFRESH-I [20], which did not show a higher incidence of adverse events with CytoSorb^®^ intraoperative hemoadsorption. Ongoing market surveillance since its initial CE mark approval over a decade ago has not identified any unanticipated device-related adverse events. Moreover, the findings of a recent meta-analysis on RCTs involving critically ill patients indicated that there was no increased risk of adverse events associated with CytoSorb treatment [42]. As the adsorption process is concentration-dependent [43], clinically meaningful removal occurs only when the plasma concentration of the target molecule is substantially elevated. A recent, prospective RCT in healthy volunteers demonstrated definitively the ability of the device to remove cytokines, with no signs of long-term immune system suppression by the treatment [44]. In addition to reducing elevated levels of cytokines, there is evidence of concurrent removal of trigger molecules, such as PAMPs (pathogen-associated molecular patterns) and DAMPs (damage-associated molecular patterns) [45] which also reside in the above-described adsorption range (Fig. 4). In this way, CytoSorb^®^ therapy aims to help the patient’s body mitigate the cytokine hyper-release cytotoxic effect and attenuate the dysregulated inflammatory response, to prevent the progression of organ dysfunction.

### Interpretation of the available evidence

Hemoadsorption represents a reasonably new technology and only a few relatively small RCTs exist. The intraoperative use of CytoSorb^®^ by direct integration in the CPB circuit was first reported by Born et al. [46] in 2014 and showed significant reductions of interleukin (IL)-6 and procalcitonin (PCT). The first RCT in patients undergoing elective cardiac surgery was published 2 years later [15], but in contrast, it did not demonstrate the removal of measured cytokines. Given the trial’s results, and as described in the elective cardiac surgery part of this review, it seems that patients undergoing elective, low-risk cardiac surgery may not be the most relevant cohort for hemoadsorption to demonstrate any effect. While the RCTs included in this systematic review confirm that the intraoperative integration of CytoSorb^®^ in CPB during cardiac surgery is both easy and safe, they mostly failed to show significant improvements in clinical endpoints, despite reducing circulating cytokine levels [10].

Considering the fact that hemoadsorption occurs in a concentration-dependent manner [43] and that effective removal requires highly increased circulating levels, it is understandable that hemoadsorption may not provide substantial clinical benefits in low-risk, elective cardiac surgery [15, 21], or even in lower-risk infective endocarditis (IE) patients [8], where cytokine concentrations are not substantially elevated.

#### Infective endocarditis

Over one-third of all publications identified in this review report on outcomes with hemoadsorption in IE patients undergoing cardiac surgery (Table 1). The results are not uniform, and their interpretation commands a thorough assessment. The REMOVE trial (Revealing Mechanisms and Investigating Efficacy of Hemoadsorption for Prevention of Vasodilatory Shock in Cardiac Surgery Patients with Infective Endocarditis—a Multicentric Randomized Controlled Group Sequential Trial) was the most extensive study within this group [10]. In a proof-of-concept pre-specified analysis in the first 50 enrolled patients, the REMOVE investigators showed a significant reduction of various cytokines. Based on these findings, they continued with full enrolment in the trial which ultimately did not show a statistically significant difference in the primary endpoint of postoperative organ function improvement assessed by the change in SOFA score between 138 patients who received intraoperative hemoadsorption and 144 patients without this treatment. All secondary outcomes and rates of adverse events were also comparable between the two groups. The patient population comprised of “all-comers” with IE requiring surgery with no further risk-stratification for inclusion except a European System for Cardiac Operative Risk Evaluation Score—EuroSCORE II > 3%. Approximately 1/3 of procedures were outpatients undergoing elective surgery, and, e.g., the median preoperative IL-6 levels were only 18.36 pg/mL in the treatment and 40.6 pg/mL (p = 0.3) in the control group. The results of REMOVE should be critically taken into account, however this trial has ultimately proven CytoSorb’s safety, but also the efficacy of cytokine adsorption, together with the more recent highly standardized and reproducible experimental human endotoxemia model of systemic inflammation and immunological tolerance [44].

Results from two other smaller RCTs in IE patients yielded different results. Asch et al. [8] found higher vasopressor and fluid therapy demand within the intervention group. In contrast, Holmen et al. [12] showed a reduction in the consumption of blood products and a substantial decrease in norepinephrine need and postoperative vasopressor therapy duration associated with CytoSorb^®^ treatment. While patient selection in the first study was compromised with significant baseline differences between the groups (Table 1), the second study included only acute, high-risk patients and demonstrated a clear trend towards faster hemodynamic stabilization correlating with hemoadsorption. Nevertheless, the minimal sample size of both trials requires caution with interpretations and conclusions.

Kalisnik et al. [13] compared 99 high-risk IE patients with intraoperative hemoadsorption to 99 propensity-score-matched controls with a median EuroSCORE II of around 9% in both groups. Similarly, Haidari et al. [11] reported on a cohort of 70 patients with a EuroSCORE II > 8%, therefore including only high-risk patients. Both studies showed a significantly lower incidence of sepsis-related mortality using hemoadsorption. In addition, the former showed a lower incidence of sepsis, and the latter reduced cumulative vasopressor need in patients treated with CytoSorb^®^. In contrast, a study by Santer et al. [9], showed that intraoperative hemoadsorption was associated with increased vasopressor and blood product requirements in 41 IE patients compared with 200 matched controls. Notably, the authors of this study acknowledged that, despite utilizing advanced statistical methods to mitigate residual confounders, there were significant disparities between the two groups, especially regarding changes in treatment protocols over time.

The overall incidence of IE is increasing [47, 48] and around 50% of these patients will require valve surgery at some point [49]. Many patients will require emergency surgery under active infection and concomitant inflammation. In such cases, major surgical trauma and CPB may worsen the already primed and aggravated immune system, often leading to excessive release of cytokines and consequent systemic hyperinflammation [50]. Moreover, the proportion of IEs caused by *Staph. aureus* has increased in recent years [48], introducing even more complexity and risk within this field. This sub-population of IE patients was investigated explicitly in a recent dual-center study in which the use of hemoadsorption was associated with significantly lower VIS, incidence of renal failure requiring dialysis, and fewer deaths (sepsis-related, 30-day, and 90-day mortality) [14]. In addition, certain bacteria toxins may also be removed by CytoSorb^®^ [45], specifically *Staph. aureus* exotoxins. This fact and the promising results from the above observational studies potentially promote CytoSorb^®^ as an adjunctive and versatile tool in high-risk IE cases.

The benefit of non-selective depletion of cytokines has not yet been proven in this population of patients. Notwithstanding, based on the results of studies presented here, it seems that hemoadsorption may improve outcomes in high-risk IE patients [5, 6, 11–14]. This is supported by the high baseline risk scores (EuroSCORE-II > 8%) [51] in the studies that reported benefit with hemoadsorption. Therefore, appropriate patient selection at high risk for postoperative complications, relating to a heightened inflammatory response, is critical when considering the use of hemoadsorption. For example, Haidari et al. [7] showed favorable outcomes after carefully selecting patients to receive intraoperative hemoadsorption based on the presence of the following criteria prior to surgery: fever, severely elevated inflammatory parameters, and/or hemodynamic instability requiring high inotropic support. Moreover, Kühne et al. [6] suggested that IE patients who, despite receiving hemoadsorption treatment during CPB, develop intraoperative renal failure and require increasing vasopressor therapy, or have high-grade intraoperative findings (vegetations and aortic root abscess), might benefit from the continuation of hemoadsorptive therapy in the ICU. Accordingly, REMOVE’s “neutral” results may potentially be reflective of the “all-comer” nature of the population [10], which is also supported by the low median IL-6 levels prior to surgery. In contrast, the average IL-6 levels seen by Jansen et al. [44] were above 500 pg/mL and demonstrated significant cytokine clearance with CytoSorb^®^.

#### Aortic surgery

Complex and combined open aortic surgery and high-risk operations to treat aortic dissections or thoracoabdominal aortic aneurism (TAAA), may also result in systemic hyperinflammation. Hypothermic circulatory arrest (HCA), commonly employed during aortic surgery extending into the arch, may be a further trigger for an exaggerated inflammatory response, often leading to vasoplegia, compromised microcirculation, increased lactate levels, and subsequent organ failure [52].

Saller et al. [25] observed significantly lower norepinephrine concentrations and better acid–base status (reflected by less frequent low pH, lower lactate concentrations, and decreased need for buffer solution) compared to standard therapy in 168 patients who underwent various open thoracic aortic surgical procedures under HCA with intraoperative CytoSorb^®^ treatment compared to 168 propensity score-matched controls. Interestingly, hemoadsorption correlated with a significantly decreased need for transfusion of packed red blood cells and fresh frozen plasma but an increased requirement of prothrombin complex concentrate. The authors observed that the overall benefit of the therapy was explicitly prominent in the subgroup of emergency patients with acute aortic dissections. A significant reduction in norepinephrine and IL-6 in patients who mainly underwent Bentall procedures adjunctly treated with CytoSorb^®^ on CPB was also shown in a small pilot observational study, together with better mean arterial pressure, and PaO_2_/FiO_2_ ratio, shorter mechanical ventilation duration, and ICU and hospital stay [26]. A pilot RCT investigated the feasibility and effect of intraoperative hemoadsorption during open TAAA repair and showed a significantly lower incidence of ARDS [27]. However, both these pilot study results should be interpreted with caution due to their small sample size.

The available evidence in the population of complex aortic surgery seems promising but remains very preliminary and requires confirmation in prospective trials. Decreased vasopressor requirements observed in high-risk aortic surgery patients who received CytoSorb^®^ therapy are similar to findings within other populations discussed in this review and may represent a meaningful clinical endpoint for future trials.

#### Post-cardiac surgery complications

Data from sizeable cardiac surgery registries show a downward trend in mortality and morbidities after cardiac surgery over the last 20 years. However, despite decades of innovation in cardiopulmonary support, on-pump cardiac surgery still carries the risk for a postoperative systemic inflammatory response and vasoplegia which in turn leads to worse outcomes [53]. In this fairly heterogeneous patient population, hemoadsorption is frequently used in daily practice to attenuate the post-operative hyperinflammatory response.

To the best of our knowledge, no prospective trials have been conducted in this population so far, and the four studies in this review provide promising but highly speculative results, involving patients with predominantly SIRS, multiorgan failure (MOF), cardiogenic and septic shock. Almost all patients required renal or circulatory support, thus CytoSorb^®^ was used adjacent to CRRT or vaECMO. It seems that hemoadsorption was associated with hemodynamic stabilization and lower actual *versus* expected mortality.

The versatile nature of hemoadsorption may benefit postoperative patients in complex and severely impaired conditions, as CytoSorb^®^ not only removes cytokines, but also bilirubin, bile acids, myoglobin, some toxins, and various PAMPs and DAMPs [45, 54–56]. As with any other blood purification technology, hemoadsorption carries a risk of inadvertent drug removal. Critical care patients are specifically prone to this due to the usually high numbers of administered medications over long treatment periods. Assessment of the clinical relevance of potential drug removal requires consideration of the patient’s condition, the impact of concomitantly applied extracorporeal therapies, duration of device exposure, and timing of drug administration. Clinical decision-making regarding adjustments in drug dosing should always be made in the broader clinical context supported by therapeutic drug monitoring when available [57].

#### Heart transplant surgery and Ex vivo organ perfusion

One study suggested favorable outcomes in HTx patients associated with intraoperative hemoadsorption [28], and an animal experiment in ex vivo lung perfusion (EVLP) showed that CytoSorb^®^ treatment significantly decreased cytokine levels and levels of immune cells post-transplantation. Histology demonstrated fewer signs of lung injury and primary graft dysfunction (PGD) incidence was significantly reduced among treated animals [58]. Authors suggest this treatment will increase the availability of the donor’s lungs and provide better graft tolerability in the recipient. The first-in-human (micro)study published by the same group [59] suggests that cytokine adsorption adjacent to extracorporeal lung support during lung transplantation supports graft acceptance. Promising results in an EVLP animal model were previously reported by Iskender et al. [60] and in animal studies involving hearts and kidneys donated after circulatory death (DCD) [61, 62]. Considering the ongoing unmet need for organs for transplantation, ex vivo organ perfusion with adjunctive hemoadsorptive treatment may play an essential role in combatting organ shortage and early graft rejection.

The use of hemoadsorption in organ transplants has been controversial due to the already discussed potential for unwanted drug removal, specifically immunosuppressants. However, a detailed investigation in a large animal model reassuringly reported a minimal level of removal with frequently used immunosuppressant regimens [63]. A very recent RCT [64] confirmed CytoSorb^®^ did not affect levels of mycophenolic acid, used to prevent organ transplant rejection, and found that concentrations were comparable to the control group at all pre-defined time points. There was also no increase in the frequency of early cardiac allograft rejection in the intervention group. In their proof-of-concept trial, Nemeth et al*.* compared the effect of intra-operative CytoSorb^®^ use to standard care in 55 orthotopic heart transplantation patients (30 CytoSorb^®^ and 25 standard care). Results showed that the CytoSorb^®^ group had significantly lower vasoactive-inotropic scores (p = 0.046), a 6.4-fold decrease in the odds of developing vasoplegic syndrome (*p* = 0.028), lower PCT levels, shorter duration of mechanical ventilation hours (*p* = 0.025), and ICU (*p* = 0.022). Patients in the CytoSorb^®^ group also had lower rates of acute kidney injury (*p* = 0.004), renal replacement therapy (*p* = 0.037) and more stable hepatic bilirubin excretion. Furthermore, 30-day mortality and 1-year survival did not differ between groups. There were no reported device-related adverse events during the study period.

#### LVAD

LVAD implantation in patients with advanced heart failure carries a substantial risk of a dysregulated inflammatory response mediated by exaggerated cytokine production. Since hemoadsorption has recently yielded promising results in high-risk patients undergoing cardiac surgery by immunomodulation and consequent attenuation of over-shooting inflammation [65], the rationale for its utilization during CPB-assisted LVAD implantation surgery was investigated. However, results from the only study on this topic found in the current literature search demonstrated a significantly increased incidence of respiratory failure in the CytoSorb^®^ group compared to propensity score-matched controls. Consequently, the need for prolonged mechanical ventilation and tracheostomy was also increased with hemoadsorption. Furthermore, in-hospital mortality was notably lower in the control group [29].

The ongoing RCT CytoSorb^®^ Modulation of Surgical Inflammation During LVAD Insertion (CYCLONE-LVAD) will evaluate the role of hemoadsorption in this field (ClinicalTrials.gov Identifier: NCT04596813).

#### ECMO/ECLS

Extracorporeal life support (ECLS) for patients with severely compromised circulation via vaECMO is known to provoke a complex inflammatory reaction. This innate immune response, if severe, may lead to disrupted microcirculation, and end-organ dysfunction. Despite dramatic technological improvements with newer ECMO platforms, systemic hyperinflammation remains a relevant clinical concern [66]. Apart from the widely known pathophysiological mechanism of artificial surface contact-mediated coagulation, platelet, and complement system activation, and consequent endothelial injury, another potent trigger for cytokine hyperproduction is the release of endotoxins in response to translocation of bacteria from ischemic gut mucosa into the bloodstream [67]. If such an already explosive immune response is dysregulated, and instead of self-limiting, the cytokine storm becomes auto-amplifying, it may lead to a vicious circle and eventually death.

Cytokine adsorption has recently been introduced as an adjunctive tool to limit the hyperinflammatory response to ECMO. Clinical evidence so far is limited and controversial. It has been recommended that parameters for appropriate patient selection should include signs of shock, high vasopressor requirements, elevated lactate levels, and/or elevated IL-6, lactate, bilirubin, or myoglobin plasma levels. Potential examples where hemoadsorption may be considered include profound shock on ECMO, post-cardiotomy ECMO in patients with infection, and ECMO in the context of organ donation [68].

A recent study from Soltesz et al. [69] evaluated the impact of vaECMO-integrated hemoadsorption on the reversal of multiorgan and microcirculatory dysfunction and early mortality of refractory cardiogenic shock patients. Among 29 *vs.* 29 propensity score-matched patients, CytoSorb^®^ treatment resulted in significantly lower VIS, lactate levels, and ECMO-associated bleeding complications. Hemoadsorption was used continuously for 72 h with vaECMO therapy in those with persistent hemodynamic instability. Well-designed, prospective trials will be necessary to answer complex questions regarding the right timing for adjunctive hemoadsorption therapy, optimal duration, and proper patient selection.

### Safety and other systematic findings

This review assessed the safety of CytoSorb^®^ therapy based on the incidence of reported unanticipated device-related adverse events (UADE). Among studies that reported adverse events, there were no UADE noted. It appears, therefore, that CytoSorb^®^ has a favorable safety profile when used in cardiac surgery patients (Evidence table, Additional file 2).

**Fig. 5:**
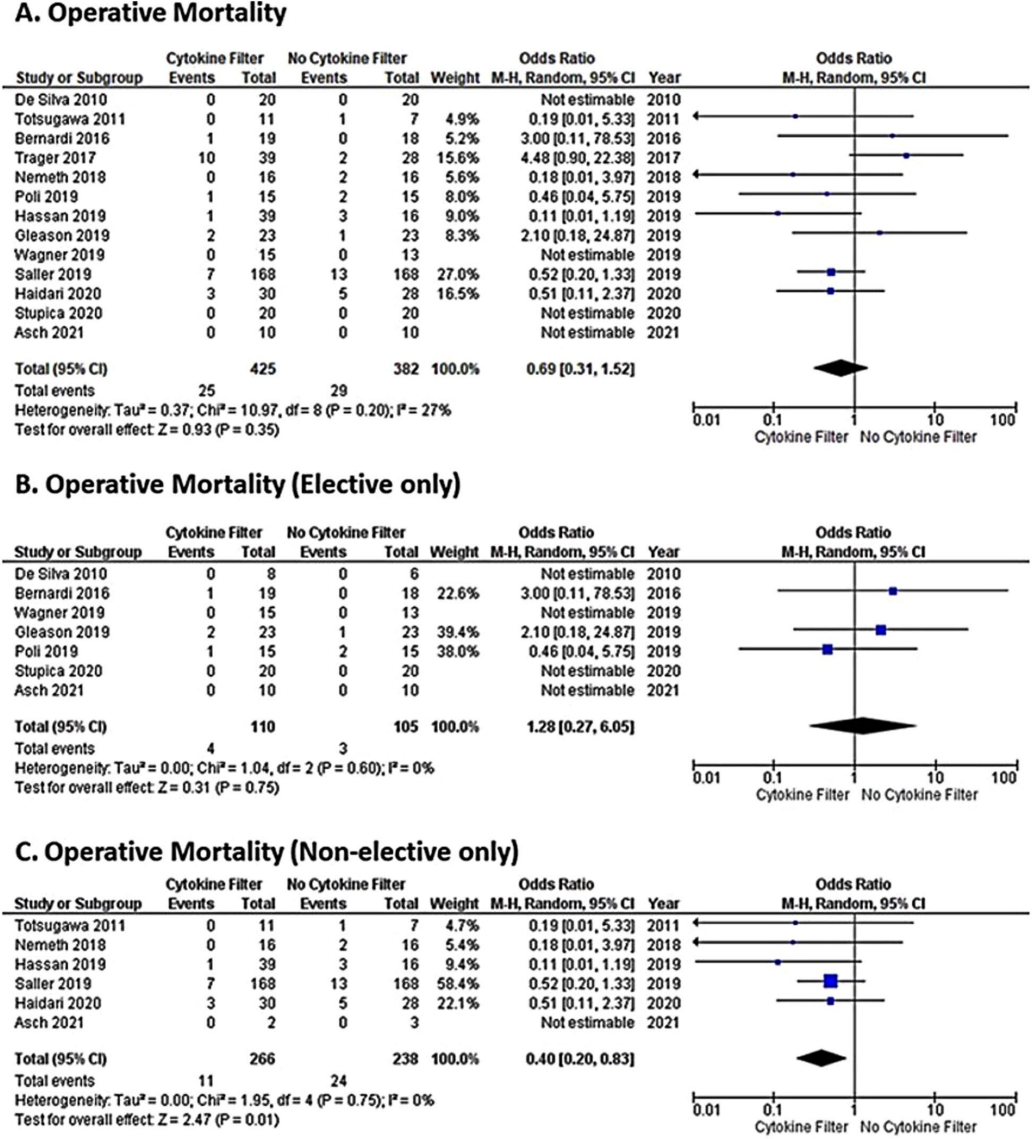
Naruka et al. [70] Forest plot: meta-analysis for the difference in operative mortality between CPB-assisted cardiac surgery with and without hemoadsorption

**Fig. 6:**
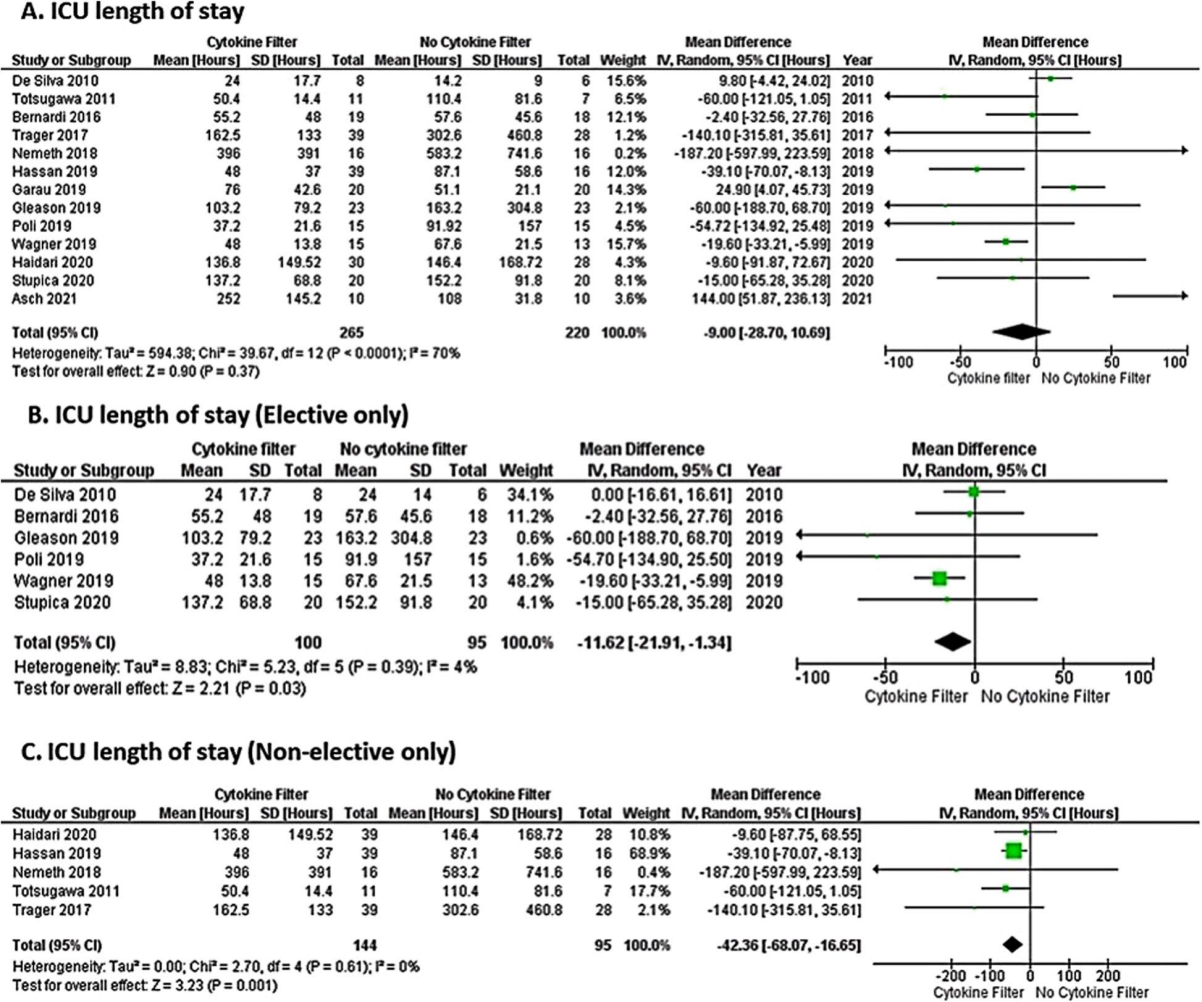
Naruka et al. [70] Forest plot: meta-analysis for the difference in an intensive care unit (ICU) stay between CPB-assisted cardiac surgery with and without hemoadsorption

A recent meta-analysis from the United Kingdom investigated operative mortality, ventilation duration, ICU and hospital stays, and postoperative day 1 inflammatory markers in studies involving CPB and hemoadsorption. Of 15 selected studies, 12 used CytoSorb^®^, 2 investigated Alteco^®^ LPS adsorber (Alteco Medical AB, Lund, Sweden), and 1 Toraymyxin^®^ (Toray Industries, Tokyo, Japan). When comparing cytokine adsorption cases and controls across all studies, authors found no significant difference in operative mortality, ventilation duration, hospital stay, and ICU length of stay. However, a significant reduction in 30-day mortality (Fig. 5) and ICU stay (Fig. 6) was shown to be associated with hemoadsorption therapy during non-elective cardiac surgery, especially emergency surgery, and in patients with a higher inflammatory burden such as with infective endocarditis [70].

The authors’ conclusions that hemoadsorption devices are likely to be more beneficial in patients with higher inflammatory responses, such as infective endocarditis and emergency operations, align with the findings of the current systematic review. Furthermore, Liu et al. [2] in their narrative review concluded that although data on the use of hemoadsorption in cardiac surgery is scarce and even controversial, there is no denying that adsorptive extracorporeal blood purification technology, especially CytoSorb^®^, opens a new door for the ongoing fforts in battling CPB-associated SIRS. A meta-analysis from Heymann et al. [42], however, showed that in fact, the use of CytoSorb® might increase mortality in critically ill patients with inflammatory conditions, although the authors did acknowledge the low certainty of the evidence, primarily due to the lack of power to independently assess mortality across the vasty heterogeneous populations included. On the other hand, the same meta-analysis reported that in critically ill patients the risk of adverse events was not higher with CytoSorb treatment. Interestingly, in a subgroup analysis of complex cardiac surgery, increased mortality associated with CytoSorb^®^ was not found, which is in agreement with the findings of Naruka et al. [70] and Liu et al., [2] as well as with the current systematic review. In general, leading experts in intensive care medicine are increasingly calling for new realistic clinical endpoints instead of mortality for the assessment of potential clinical benefits of novel therapies in critical patients [71–73].

Based on the current best practice, intraoperative hemoadsorption is applied in long-lasting, high-risk, and complex procedures with a minimum expected pump-run of at least 75–90 min. These suggested CPB cut-off duration times are currently investigated in two pivotal, double blind, randomized trials RECCAS (Removal of cytokines during cardiac surgery, German Clinical Trials Register number: DRKS00007928) and REMOTE (Removal of Cytokines in Patients Undergoing Cardiac Surgery With CPB, ClinicalTrials.gov identifier: NCT03104179).

Hemoadsorption is an adjunct therapy in critically ill or high-risk surgical patients. As such, it represents a small piece in a very complex puzzle of medical treatments, therapeutic protocols, surgical procedures and techniques, and advanced diagnostics currently used in the cardiac surgery setting and beyond. Although solid clinical evidence demonstrating survival benefits from using hemoadsorption in cardiac surgery is currently lacking, this systematic literature review, despite its limitations regarding the heterogeneity of study designs and endpoint measures, suggests that meaningful outcomes such as faster hemodynamic stabilization may be achieved with hemoadsorption in high-risk cardiac surgery patients which has also been proven by the meta-analysis of Naruka et al*.* [70].

## Conclusions

Hemoadsorption in cardiac surgery is an emerging field with CytoSorb® being the only available device with published evidence discovered in this systematic review. The reviewed evidence shows that its use intraoperatively with CPB or postoperatively with CRRT or vaECMO is feasible and safe with no unanticipated device-related adverse events reported in any of the retrieved publications. In relation to the evidence supporting the efficacy of the device, the available evidence is mixed, but in aggregate suggests limited value with its use in routine elective surgery and low-risk patients, including “cold” infective endocarditis-related valve surgery. On the other hand, hemoadsorption with on-pump cardiac surgery seems to be an effective adjunctive therapy at least in high-risk, acute or “hot” infective endocarditis cases, especially presenting with *Staphylococcus aureus* infection, and possibly in aortic surgery cases and among patients who develop a dysregulated inflammatory response, vasoplegia or septic shock postoperatively. The beneficial effect of adjunctive hemoadsorption especially in “non-elective” patients has been reported by a previously published meta-analysis. The most frequently reported clinical benefit associated with hemoadsorption treatment is reduced vasopressor demand resulting in better hemodynamic stability. CytoSorb^®^ also represents a promising new approach within the field of heart transplantation and ex vivo organ perfusion, where in addition to improved outcomes it may also contribute to greater organ availability. Further prospective (randomized controlled) studies are needed to enhance the body of evidence for the potential benefits associated with the use of hemoadsorption in cardiac surgery-related settings.

### Supplementary Information

Additional file 1. PRISMA 2020 Checklist.

Additional file 2. Evidence table.

## Data Availability

No datasets were generated or analyzed during the current study. The data used to support the findings of this review are available within the manuscript and supplementary material.
